# High-quality draft genome sequence of *Aquidulcibacter paucihalophilus* TH1–2^T^ isolated from cyanobacterial aggregates in a eutrophic lake

**DOI:** 10.1186/s40793-017-0284-9

**Published:** 2017-12-02

**Authors:** Haiyuan Cai, Yonghui Zeng

**Affiliations:** 1 State Key Laboratory of Lake Science and Environment, Nanjing Institute of Geography and Limnology, Chinese Academy of Sciences, Nanjing, China; 2 Aarhus Institute of Advanced Studies & Department of Environmental Science, Aarhus University, Aarhus, Denmark

**Keywords:** *Aquidulcibacter paucihalophilus*, Cyanobacterial aggregates, Carbohydrate active enzyme, Peptidase, Transporter

## Abstract

*Aquidulcibacter paucihalophilus* TH1–2^T^ is a member of the family *Caulobacteraceae* within *Alphaproteobacteria* isolated from cyanobacterial aggregates in a eutrophic lake. The draft genome comprises 3,711,627 bp and 3489 predicted protein-coding genes. The genome of strain TH1–2^T^ has 270 genes encoding peptidases. And metallo and serine peptidases were found most frequently. A high number of genes encoding carbohydrate active enzymes (141 CAZymes) also present in strain TH1–2^T^ genome. Among CAZymes, 47 glycoside hydrolase families, 37 glycosyl transferase families, 38 carbohydrate esterases families, nine auxiliary activities families, seven carbohydrate-binding modules families, and three polysaccharide lyases families were identified. Accordingly, strain TH1–2^T^ has a high number of transporters (91), the dominated ones are ATP-binding cassette transporters (61) and TonB-dependent transporters (28). Major TBDTs are Group I, which consisted of transporters for various types of dissolved organic matter. These genome features indicate adaption to cyanobacterial aggregates microenvironments.

**Electronic supplementary material:**

The online version of this article (10.1186/s40793-017-0284-9) contains supplementary material, which is available to authorized users.

## Introduction

Lake Taihu is the third largest freshwater lake in China, located in the rapidly-developing, economically-important Changjiang (Yangtze) River Delta. 10.1601/nm.648 spp. often form large mucilaginous blooms in the lake due to anthropogenic nutrient over-enrichment. These bloom aggregates were composed of extracellular polymeric substances, produced via a number of approaches including excretion, secretion, sorption and cell lysis, comprising a heterogeneous polymer and mainly consisted of polysaccharides, proteins, lipids and humic substances [1]. Within the bloom, a variety of niches are created within a dense scum that can be 10–30 cm in thickness [2]. The diel shifts lead to changes in the dissolved oxygen levels with oxygen enrichment during the day and depleted at night, and with microaerobic zones present at all times within the 10.1601/nm.648 spp. blooms [3]. It is known that many heterotrophic bacteria live in association with cyanobacteria [4, 5]. To maintain the dominance of the cyanobacterial bloom, bacterial taxa within the cyanobacterial aggregates possibly catalyze the turnover of complex organic matters released by cyanobacteria, to recycle the previously-loaded nutrient sources [5].

10.1601/nm.30931 type strain TH1–2^T^ (=10.1601/strainfinder?urlappend=%3Fid%3DCGMCC+1.12979 ^T^ = 10.1601/strainfinder?urlappend=%3Fid%3DLMG+28362 ^T^) is a member of the family 10.1601/nm.1249 within 10.1601/nm.809 isolated from cyanobacterial aggregates in lake Taihu, China [6]. The genus 10.1601/nm.30930 currently includes only one cultivated strain. The sequenced genome of 10.1601/nm.30931 TH1–2^T^ will provide the genetic basis for better understanding of adaptation to cyanobacterial aggregates and ecological function during the cyanobacterial bloom.

Here, we present the genome of 10.1601/nm.30931 TH1–2^T^ with special emphasis on the genes coding for carbohydrate active enzymes and peptidases. The second focus is on genes coding for dedicated transport systems for the uptake of macromolecule decomposition products which released by cyanobacteria 10.1601/nm.648 spp., such as ATP-binding cassette transporters and TonB-dependent transporter system.

## Organism information

### Classification and features

**Fig. 1:**
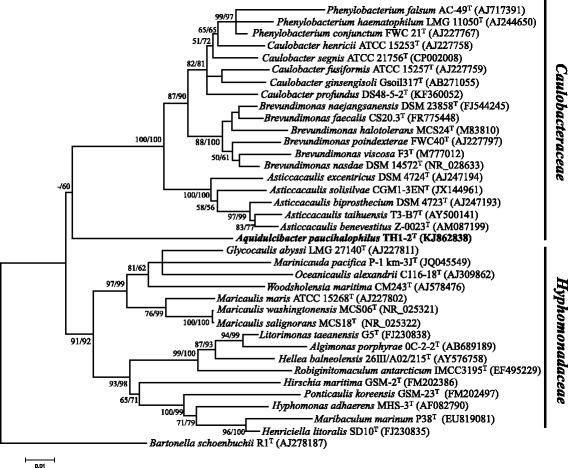
The 16S rRNA tree highlighting the position of *A. paucihalophilus* TH1–2^T^ relative to the representatives of the order *Caulobacterales* including the families *Caulobacteraceae* and *Hyphomonadaceae*. Maximum likelihood (substitution model = GTR) tree, using 1406 aligned characters, was rooted by *Bartonella schoenbuchii* R1. Branches were scaled in terms of the expected number of substitutions per site. Numbers adjacent to branches are support values from 1000 ML bootstrap replicates (left) and from 1000 maximum-parsimony bootstrap replicates (right); values below 50% were neglected

Cyanobacterial bloom samples were taken from Lake Taihu. Samples were transferred to 500 mL beakers and left at room temperature for 2 h. This resulted in flotation of the cyanobacterial aggregates to the top of the beaker. Several of the largest aggregates were selected for testing and washed three times in sterile lake water. 10.1601/nm.30931 strain TH1–2^T^ was isolated from cyanobacterial aggregates [6]. The 16S rRNA gene sequence similarities between strain TH1–2^T^ and others were <91%. The position of strain TH1–2^T^ relative to its phylogenetic neighbors is shown in Fig. 1. Strain TH1–2^T^ formed a deeply separated branch, with the genera 10.1601/nm.1263, 10.1601/nm.1266, 10.1601/nm.1250 and 10.1601/nm.1275, which belong to the family 10.1601/nm.1249, and separate from the cluster with genera of the family 10.1601/nm.14022 (Fig. 1).

**Fig. 2:**
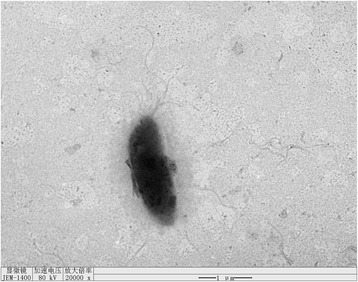
Images of *A. paucihalophilus* TH1–2^T^ using transmission electron micrograph

Cells of strain TH1–2^T^ are rod-shaped, with a length of 1.8–2.2 μm and a width of 0.8–1.1 μm (Fig. 2 and Table 1). Cells are motile by means of a single polar flagellum. TH1–2^T^ is a Gram-negative, aerobic, mesophilic bacterium with an optimal growth temperature is 30 °C and an optimal salinity is 0%. On R2A agar (Oxoid) strain TH1–2^T^ forms smooth, yellow colonies after 24 h at 30 °C. Strain TH1–2^T^ is able to utilize N-acetyl-glucosamine, citrate, gluconate, D-glucose, D-mannitol, D-maltose, phenyl acetate, L-rhamnose, and starch [6]. Strain TH1–2^T^ possesses alkaline phosphatase, esterase (C4), esterase lipase (C8), leucine arylamidase, valine arylamidase, cystine arylamidase, trypsin α-chymotrypsin, acid phosphatase, naphthol-AS-BI-phosphohydrolase, β-galactosidase, α - and β -glucosidase, and N-acetyl-β-glucosaminidase [6].

**Table 1:** Classification and general features of *A. paucihalophilus* strain TH1–2^T^ according to the MIGS recommendations [7]

MIGS ID	Property	Term	Evidence code^a^
	Classification	Domain *Bacteria*	TAS [38]
		Phylum *Proteobacteria*	TAS [39]
		Class *Alphaproteobacteria*	TAS [40]
		Order *Caulobacterales*	TAS [41, 42]
		Family *Caulobacteraceae*	TAS [42, 43]
		Genus *Aquidulcibacter*	TAS [6]
		Species *Aquidulcibacter paucihalophilus*	TAS [6]
		Type strain: TH1–2	TAS [6]
	Gram stain	negative	TAS [6]
	Cell shape	rod (1.2–2.2 μm long, 0.8–1.1 μm wide)	TAS [6]
	Motility	motile	TAS [6]
	Sporulation	none	NAS
	Temperature range	mesophile	TAS [6]
	Optimum temperature	30 °C	TAS [6]
	pH range; Optimum	7	TAS [6]
	Carbon source	N-acetyl-glucosamine, citrate, gluconate, D-glucose, D-mannitol, D-maltose, phenyl acetate, L-rhamnose, and starch	TAS [6]
MIGS-6	Habitat	Cyanobacterial aggregates in freshwater lake	TAS [6]
MIGS-6.3	Salinity	0% NaCl (*w*/*v*)	TAS [6]
MIGS-22	Oxygen requirement	aerobe	TAS [6]
MIGS-15	Biotic relationship	Cyanobacterial aggregates associated	TAS [6]
MIGS-14	Pathogenicity	unknown	NAS
MIGS-4	Geographic location	Meiliang Bay, Lake Taihu, China	TAS [6]
MIGS-5	Sample collection	2013	TAS [6]
MIGS-4.1	Latitude	31°30′N	TAS [6]
MIGS-4.2	Longitude E	120°11′E	TAS [6]
MIGS-4.3	Depth	Lake surface	TAS [6]
MIGS-4.4	Altitude	not specified	

^a^Evidence codes - TAS: Traceable Author Statement (i.e., a direct report exists in the literature); NAS: Non-traceable Author Statement (i.e., not directly observed for the living, isolated sample, but based on a generally accepted property for the species, or anecdotal evidence). These evidence codes are from the Gene Ontology project [44]

#### Chemotaxonomic data

The predominant cellular fatty acids in strain TH1–2^T^ are C_16:0_, C_16:1_ ω5c, summed feature 3 (comprising C_16:1_ ω6c and/or C_16:1_ ω7c) and summed feature 8 (consisting C_18:1_ ω6c and/or C_18:1_ ω7c). The predominant polar lipids are diphosphatidylglycerol, phosphatidylethanolamine and phosphatidylglycerol. The DNA G + C content was reported to be 55.6 mol% [6].

## Genome sequencing information

### Genome project history

10.1601/nm.30931 strain TH1–2^T^ was selected for sequencing in 2017 based on its phylogenetic position and its isolation environment [6]. The quality draft assembly and annotation were made available for public access on Apr 24, 2017. The genome project is deposited in the Genomes OnLine Database as project Gp0225845. This Whole Genome Shotgun project has been deposited at GenBank under the accession NCSQ00000000.1. The NCBI accession number for the Bioproject is PRJNA382246. Table 2 presents the project information and its association with MIGS version 2.0 compliance [7].

**Table 2:** Project information

MIGS ID	Property	Term
MIGS-31	Finishing quality	High quality draft
MIGS-28	Libraries used	Nextera XT
MIGS-29	Sequencing platforms	Illumina HiSeq PE150
MIGS-31.2	Fold coverage	1380×
MIGS-30	Assemblers	SOAPdenovo v. 2.01
MIGS-32	Gene calling method	Prodigal v2.50, IMG-ER
	Locus Tag	B7364
	Genome Database release	IMG; 2,687,453,711
	Genbank ID	NCSQ00000000.1
	Genbank Date of Release	April 24th, 2017
	GOLD ID	Gp0225845
	BIOPROJECT	PRJNA382246
MIGS-13	Source Material Identifier	TH1–2
	Project relevance	environmental

### Growth conditions and genomic DNA preparation

10.1601/nm.30931 strain TH1–2^T^ was grown in R2A agar medium at 30 °C, as previously described [6]. Genomic DNA was isolated from 0.5 g of cell paste using Gentra Puregene Yeast/Bact. Kit (Qiagen) as recommended by the manufacturer.

### Genome sequencing and assembly

Whole-genome sequencing was performed using the Illumina technology. Preparation of paired-end sequencing library with the Illumina Nextera XT library preparation kit and sequencing of the library using the Illumina HiSeq PE150 were performed as described by the manufacturer (Illumina, San Diego, CA, USA). A total of 17,033,314 paired-end reads totaling 5109.9 Mbp remained after quality trimming and adapter removal with Trimmomatic-0.33 [8]. The trimmed reads represented an average genome coverage of ~1380-fold based on the size of the assembled draft genome of strain TH1–2^T^. De novo assembly of all trimmed reads with SOAPdenovo v2.0 [9] resulted in 174 contigs. A summary of project information is shown in Table 2.

### Genome annotation

Protein-coding genes were identified as part of the genome annotation pipeline the Integrated Microbial Genomes Expert Review platform using Prodigal v2.50. The predicted CDSs were translated and used to search the National Center for Biotechnology Information non-redundant database, UniProt, TIGR-Fam, Pfam, PRIAM, KEGG, COG, and InterPro database. These data sources were combined to assert a product description for each predicted protein. Non-coding genes and miscellaneous features were predicted using tRNAscan-SE [10], RNAmmer [11], Rfam [12], TMHMM [13] and SignalP [14]. Additional gene prediction analyses and functional annotation were performed within the IMG-Expert Review platform [15].

## Genome properties

The assembly of the draft genome sequence consists of 174 contigs amounting to 3,711,627 bp. The G + C content is 55.7 mol% (Table 3). A total of 3544 genes with 3489 protein-coding genes were predicted, whereas 2758 (77.82% of total genes) protein-encoding genes were associated with predicted functions. Of the RNA, 42 are tRNAs and 3 are rRNAs. The genome statistics are further provided in Table 3. The distribution of genes into functional categories (clusters of orthologous groups) is shown in Table 4.

**Table 3:** Genome sequencing statistics of the *A. paucihalophilus* TH1–2^T^ genome

Attribute	Value	% of total
Genome Size (bp)	3,711,627	100
DNA coding (bp)	3,351,009	90.28
DNA G + C (bp)	2,065,972	55.7
Total genes	3544	100
Protein-coding genes	3489	98.45
RNA genes	55	1.55
Pseudo genes	0	0
Genes in internal clusters	621	17.52
Genes with function prediction	2758	77.82
Genes assigned to COGs	2379	67.13
Genes assigned to Pfam domains	2844	80.25
Genes with signal peptides	391	11.03
Genes with transmembrane helices	803	22.66
CRISPR repeats	105

**Table 4:** Number of genes associated with the general COG functional categories

Code	Value	% age	Description
J	189	7.12	Translation, ribosomal structure and biogenesis
A	n.a.	n.a	RNA processing and modification
K	174	6.56	Transcription
L	109	4.11	Replication, recombination and repair
B	2	0.08	Chromatin structure and dynamics
D	30	1.13	Cell cycle control, cell division, chromosome partitioning
V	68	2.56	Defense mechanisms
T	112	4.22	Signal transduction mechanisms
M	165	6.22	Cell wall/membrane/envelope biogenesis
N	48	1.81	Cell motility
U	77	2.90	Intracellular trafficking, secretion, and vesicular transport
O	132	4.97	Posttranslational modification, protein turnover, chaperones
C	138	5.20	Energy production and conversion
G	135	5.09	Carbohydrate transport and metabolism
E	188	7.08	Amino acid transport and metabolism
F	66	2.49	Nucleotide transport and metabolism
H	146	5.50	Coenzyme transport and metabolism
I	180	6.78	Lipid transport and metabolism
P	130	4.90	Inorganic ion transport and metabolism
Q	104	3.92	Secondary metabolites biosynthesis, transport and catabolism
R	235	8.85	General function prediction only
S -	177 1165	6.67 32.87	Function unknown Not in COGs

Abbreviation: *n.a.* not assigned

The total is based on the total number of protein coding genes in the genome

## Insights from the genome sequence

### Energy metabolism

10.1601/nm.30931 TH1–2^T^ has the complete Embden-Meyerhof-Parnas pathway, pentose 5-phosphate pathway and Entner-Doudoroff Pathway. For pyruvate oxidation to acetyl-coenzyme A, TH1–2^T^ contains a three-component pyruvate dehydrogenase complex. TH1–2^T^ has a complete tricarboxylic acid cycle with the glyoxylate shunt and a redox chain for oxygen respiration, including a sodium-transporting NAD(H): quinone oxidoreductase (complex I), succinate dehydrogenase (complex II), cytochrome c type (complex IV) terminal oxidases, and a F0F1-type ATPase. The complex III (cytochrome bc1) is absent. Under anoxic conditions, TH1–2^T^ has the potential for a mixed acid fermentation, such as acetyl-coA fermentation to butyrate, as indicated by presence of a 3-hydroxybutyryl-CoA dehydrogenase. TH1–2^T^ likely stores energy and phosphorus in the form of polyphosphate, since the genome encodes an exopolyphosphatase and a polyphosphate kinase.

10.1601/nm.30931 TH1–2^T^ is able to grow on organic acid, amino acid, and various sugar [6]. Based on COG functional categories (Table 4), The majority of genes of 10.1601/nm.30931 associated with translation, ribosomal structure and biogenesis, amino acid transport and metabolism, lipid transport and metabolism, transcription, cell wall/membrane/envelope biogenesis, coenzyme transport and metabolism, energy production and conversion, and carbohydrate transport and metabolism of which the proportions were higher than 5%. The high number of proteins in these classes indicated that 10.1601/nm.30931 TH1–2^T^ possessed a delicate regulation system as well as a requirement for sufficient organic in its lifestyle.

Comparison of different functional categories with other model bacteria (10.1601/nm.3093 K12 [16], 10.1601/nm.2674 KT2440 [17], 10.1601/nm.2931 MR-1 [18] revealed remarkable differences in the distribution of functional categories of predicted proteins (Additional file 1: Table S1). 10.1601/nm.30931 TH1–2^T^ had the highest proportion of genes devoted to lipid metabolism, which was even higher than that of 10.1601/nm.2674 KT2440 (4.01%), an important environmental bacterium involved in biodegradation. From the genes assigned to lipid metabolism, 33 genes were related to fatty acid degradation based on KEGG database. 10.1601/nm.30931 TH1–2^T^ also had an increased proportion of coenzyme transport and metabolism, carbohydrate transport and metabolism, and protein turnover. The distinctive percentage of genes for various metabolisms indicated that 10.1601/nm.30931 TH1–2^T^ had sophisticated systems to uptake and metabolize lipid, carbohydrate, and protein. This provides clues to different roles of 10.1601/nm.30931 strain TH1–2^T^ in cyanobacterial aggregates environments.

### Carbohydrate active enzymes

10.1601/nm.30931 TH1–2^T^ was isolated from cyanobacterial aggregates, hydrolyzes casein, starch and hemicellulose [6]. Therefore, we compared the predicted CDS against the CAZyme and dbCAN [19] database. The genome of strain TH1–2^T^ comprised a high number and high diversity of carbohydrate active enzymes including a total of 47 glycoside hydrolases, 37 glycosyl transferases, 38 carbohydrate esterases, 9 auxiliary activities, 7 carbohydrate-binding modules, and 3 polysaccharide lyases (Table 5).

**Table 5:** CAZyme profile of *A. paucihalophilus* TH1–2^T^

CAZy family	AA2	AA3	AA4	AA6	AA7		CBM4	CBM48
Counts	1	3	2	1	1		1	3
CAZy family	CBM50		CE1	CE3	CE4	CE9	CE10	CE11
Counts	1		12	2	5	2	15	1
CAZy family	CE15		GH3	GH5	GH13	GH15	GH16	GH23
Counts	1		4	2	8	1	1	9
CAZy family	GH24	GH36	GH42	GH43	GH53	GH63	GH68	GH77
Counts	1	1	1	1	1	1	1	1
CAZy family	GH84	GH92	GH97	GH102	GH103	GH109	GH130	GH133
Counts	2	1	1	1	1	4	2	1
CAZy family		GT2	GT4	GT9	GT19	GT26	GT27	GT28
Counts		14	10	1	1	1	1	1
CAZy family	GT30	GT51	GT66	GT81	GT83		PL1	PL22
Counts	1	4	1	1	1		2	1

The 10.1601/nm.30931 TH1–2^T^ genome encodes CAZymes with expected properties such as peptidoglycan synthesis and remodelling/degradation (belonging to GT28 and GT51 families and GH3, GH23, GH24, GH102 and GH103 families respectively), and lipopolysaccharide biosynthesis pathway (belonging to GT9, GT19, GT30, GT83 families). Furthermore, 10.1601/nm.30931 TH1–2^T^ has the potential to produce glucose from glycogen by candidate α-amylases belonging to GH13 family (eight in total). In addition, there were also other two cellulase classes for the complete degradation of hemicellulose by endo-1,4-β-mannosidase of families GH5 (2 copies) and β-glucosidase of families GH3 (4 copies).

Members of families CE1 and CE10, represented a significant proportion (71%) of the total CEs, share the common activities of carboxylesterase and endo-1,4-β-xylanase [20]. However, they have a great diversity in substrate specificity. For example, vast majority of CE10 enzymes act on non-carbohydrate substrates [21]. Out of the 12 GT families identified in TH1–2^T^ genome, enzymes belonging to families GT2 and GT4 (cellulose synthase, chitin synthase, α-glucosyltransferase, etc.) represented a significant proportion (64%) of the total GTs.

Lignin-degrading enzymes of which, CAZyme families AA3 (glucose/methanol/choline oxidoreductases) and AA7 (glucooligosaccharide oxidase) appeared to be present in strain TH1–2^T^ genome (Table 5). The family AA3 enzymes provide hydrogen peroxide required by the family AA2 enzymes (class II peroxidases) for catalytic activity, whereas family AA7 enzymes are known to be involved in the biotransformation or detoxification of lignocellulosic biomass [22]. Generally, the families AA1 enzymes (multicopper oxidase) and AA2 enzymes (class II peroxidase) are the main oxidative enzymes that degrade phenolic and non-phenolic structures of lignin.

Pectate lyases PL1 (2 copies) possessed in this strain suggested that these enzymes could degrade pectin associated with cyanobacteria. CBMs which have no reported enzymatic activity on their own, but can potentiate the activities of all other CAZymes (GHs, CEs, and auxiliary enzymes) or act as an appendix module of CAZymes [23, 24].

### Peptidases

The MEROPS annotation was carried out by searching the sequences against the MEROPS 12.0 database [25] (access date: 2017.10.16, version: pepunit.lib) as described in Hahnke et al. [26]. The genome of strain 10.1601/nm.30931 TH1–2^T^ comprised 270 identified peptidase genes (or homologues), mostly serine peptidases (S, 133), metallo peptidases (M, 56) and cysteine peptidases (C, 27) (Table 6). Among serine peptidases, members of the families S09 and S33, both of which cleave mainly prolyl bonds [27], are most prevalent in 10.1601/nm.30931 TH1–2^T^. S09 members act mostly on oligopeptides, probably due to the confined space in the N-terminus of their β-propeller tunnel [28, 29], and S33 members release an N-terminal residue from a peptide, preferably (but not exclusively) a proline [28]. So far, S9 and S33 peptidases have been connected to the degradation of proline-rich proteins from animals [30–32] and are not known for a role in the biodegradation of algal biomass.

**Table 6:** Peptidases and simple peptidase inhibitors in the genome of *A. paucihalophilus* TH1–2^T^

Peptidase	A08	A24	A28		C09	C13	C26	C39
Counts	1	1	1		1	1	13	1
Peptidase	C40	C44	C56	C82	C93	C96		M01
Counts	1	5	1	2	1	1		3
Peptidase	M03	M13	M14	M15	M16	M17	M19	M20
Counts	2	1	2	1	4	2	1	7
Peptidase	M23	M24	M28	M38	M41	M48	M50	M79
Counts	12	3	2	8	1	3	2	1
Peptidase	M96		N06	N11		P01		S01
Counts	1		1	1		1		8
Peptidase	S06	S08	S09	S11	S12	S14	S16	S24
Counts	1	3	35	2	15	2	5	1
Peptidase	S26	S29	S33	S41	S45	S46	S49	S54
Counts	5	1	36	2	3	1	12	1
Peptidase		T01	T02		T03	T05		U32
Counts		1	2		4	1		3
Peptidase	U62	U73						
Counts	2	2						
Inhibitor	I39	I42	I71	I87				
Counts	27	1	1	4				

Among the present metalloproteinases, members of the families M23 belong to the most frequent ones. M23 family members have been shown to take part in the extracellular degradation of bacterial peptidoglycan, either as a defense or as a feeding mechanism [33]. The complete extracellular decomposition of peptides to amino acids requires M20 and M28 family exopeptidases [27], both of which can be found abundantly in the 10.1601/nm.30931 TH1–2^T^ genome as well.

### Transport systems

Sixty-one ATP-binding cassette transporters, one tripartite ATP-independent periplasmic transporters, one phosphotransferase system transporters, 28 TonB-dependent transporters were identified in TH1–2^T^ genome. ABC transporters are ubiquitous in bacteria and function in the import of growth substrates or factors, including carbohydrates, amino acids, polypeptides, vitamins, and metal-chelate complexes [34]. TBDT in the bacterial outer membrane often promotes the transport of rare nutrients and is known for its high-affinity uptake of iron complexes. Experimental data reveal that carbohydrates, amino acid, and organic acid are TonB-dependent substrates [35, 36]. Twenty-eight TBDTs detected in TH1–2^T^ genome were classified by aligning these genes with genes within different clusters classified by Tang et al., [37]. Group I TBDTs, which was dominated in TH1–2^T^ genome, consisted of transporters for various types of dissolved organic matter, including carbohydrates, amino acids, lipids, organic acid, and protein degradation products (Table 7). Nine genes were identified as group III TBDTs, that transport iron from heme or iron proteins with high affinity (Table 7). Thirty-seven genes were related to porphyrin and chlorophyll metabolism based on KEGG database.

**Table 7:** TBDTs in the genome of *A. paucihalophilus* TH1–2^T^

Function categories	Cluster number	Gene number	Substrates
Group I: DOM transporters	Cluster 3090	5	Chito-oligosaccharides, phytate, maltodextrin, maltose, chitin, xylan, xylose, pectin
	Cluster 427	4	Arabinose
	Cluster 952	4	Sucrose
Group II: Siderophores/Vitamins transporters	Cluster 410	1	siderophore
	Cluster 973	3	Vitamin B12, catecholates, enterobactin, 2,3-dihydroxybenzoylserine (DHBS)
Group III: Heme/Hemophores/ Iron(heme)-binding transporters	Cluster 1586	9	Heme
Group IV: Metal transporters	Cluster 767	2	Copper, Copper chelate

## Conclusions

The genome of 10.1601/nm.30931 TH1–2^T^ contains a relatively high number of genes coding for fatty acid degradation, carbohydrate active enzymes and peptidase, and transporter. The availability of 10.1601/nm.30931 TH1–2^T^ draft genome sequence may provide better insights into its primary metabolism and other phenotypic characteristics of interest. Further studies involving characterization of carbon element cycling genes would accentuate its biogeochemical cycling importance, particularly in ecological restoration for the eutrophic lake.

## Additional file

Additional file 1: Table S1. comparison of proportions of COG categories between A. paucihalophilus TH1–2 T, *E. coli* K12, P. putida KT2440, and S. oneidensis MR-1. (DOCX 13 kb)

## Abbreviations

AA: Auxiliary activities
ABC: ATP-binding cassette
CBM: Carbohydrate-binding modules
CE: Carbohydrate esterases
DOM: Dissolved organic matter
ED: Entner-Doudoroff pathway
EMP: Embden-Meyerhof-Parnas pathway
GH: Glycoside hydrolases
GT: Glycosyl transferases
IMG-ER: Integrated microbial genomes – expert review
PL: Polysaccharide lyases
PP: Pentose 5-phosphate pathway
PTS: Phosphotransferase system
TBDT: TonB-dependent transporter
TRAP: Tripartite ATP-independent periplasmic

## References

[1] Xu H, Cai H, Yu G, Jiang H (2013). Insights into extracellular polymeric substances of cyanobacterium *Microcystis aeruginosa* using fractionation procedure and parallel factor analysis. Water Res.

[2] Xing P, Guo L, Tian W, Wu QL (2011). Novel *Clostridium* populations involved in the anaerobic degradation of Microcystis blooms. ISME J.

[3] Wang H, Lu J, Wang W, Yang L, Chen Y (2006). Methane fluxes from the littoral zone of hypereutrophic Taihu Lake, China. J Geophys Res.

[4] Cai H, Yan Z, Wang A, Krumholz LR, Jiang H (2013). Analysis of the attached microbial community on mucilaginous cyanobacterial aggregates in the eutrophic lake Taihu reveals the importance of *Planctomycetes*. Microbial Ecol.

[5] Cai H, Jiang H, Krumholz LR, Yang Z (2014). Bacterial community composition of size-fractioned aggregates within the phycosphere of cyanobacterial blooms in a eutrophic freshwater lake. PLoS One.

[6] Cai H, Shi Y, Wang Y, Cui H, Jiang H (2017). *Aquidulcibacter paucihalophilus* gen. Nov., sp. nov., a novel member of family *Caulobacteraceae* isolated from cyanobacterial aggregates in a eutrophic lake. Antonie Van Leeuwenhoek.

[7] Field D, Garrity G, Gray T, Morrison N, Selengut J, Sterk P (2008). The minimum information about a genome sequence (MIGS) specification. Nat Biotech.

[8] Bolger AM, Lohse M, Usadel B (2014). Trimmomatic: a flexible trimmer for Illumina sequence data. Bioinformatics.

[9] Luo R, Liu B, Xie Y, Li Z, Huang W, Yuan J (2012). SOAPdenovo2: an empirically improved memory-efficient short-read de novo assembler. Gigascience.

[10] Lowe TM, Eddy SR (1997). tRNAscan-SE. A program for improved detection of transfer RNA genes in genomic sequence. Nucleic Acids Res.

[11] Lagesen K, Hallin P, Rodland EA, Staerfeldt HH, Rognes T, Ussery DW (2007). RNAmmer: consistent and rapid annotation of ribosomal RNA genes. Nucleic Acids Res.

[12] Nawrocki EP, Burge SW, Bateman A, Daub J, Eberhardt RY, Eddy SR (2015). Rfam 12.0: updates to the RNA families database. Nucleic Acids Res.

[13] Krogh A, Larsson B, von Heijne G, Sonnhammer EL (2001). J Mol Biol.

[14] Bendtsen JD, Nielsen H, von Heijne G, Brunak S (2004). Improved prediction of signal peptides: SignalP 3.0. J Mol Biol.

[15] Markowitz VM, Chen IM, Palaniappan K, Chu K, Szeto E, Pillay M (2014). IMG 4 version of the integrated microbial genomes comparative analysis system. Nucleic Acids Res.

[16] Blattner FR, Plunkett G, Bloch CA, Perna NT, Burland V, Riley M (1997). The complete genome sequence of *Escherichia coli* K-12. Science.

[17] Nelson K, Weinel C, Paulsen I, Dodson R, Hilbert H (2002). Martins dos Santos VA, et al. complete genome sequence and comparative analysis of the metabolically versatile *Pseudomonas putida* KT2440. Environ Microbiol.

[18] Ng CK, Sivakumar K, Liu X, Madhaiyan M, Ji L, Yang L (2013). Influence of outer membrane c-type cytochromes on particle size and activity of extracellular nanoparticles produced by *Shewanella oneidensis*. Biotechnol Bioeng.

[19] Yin Y, Mao X, Yang J, Chen X, Mao F, Xu Y (2012). dbCAN: a web resource for automated carbohydrate-active enzyme annotation. Nucleic Acids Res.

[20] Zhao Z, Liu H, Wang C, Xu JR (2013). Comparative analysis of fungal genomes reveals different plant cell wall degrading capacity in fungi. BMC Genomics.

[21] Cantarel BL, Coutinho PM, Rancurel C, Bernard T, Lombard V, Henrissat B (2009). The carbohydrate-active EnZymes database (CAZy): an expert resource for Glycogenomics. Nucleic Acids Res.

[22] Levasseur A, Drula E, Lombard V, Coutinho PM, Henrissat B (2013). Expansion of the enzymatic repertoire of the CAZy database to integrate auxiliary redox enzymes. Biotechnol Biofuels.

[23] Tomme P, Warren RAJ, Gilkes NR (1995). Cellulose hydrolysis by bacteria and fungi. Adv Microb Physiol.

[24] Boraston AB, Bolam DN, Gilbert HJ, Davies GJ (2004). Carbohydrate-binding modules: fine-tuning polysaccharide recognition. Biochem J.

[25] Rawlings ND, Waller M, Barrett AJ, Bateman A (2014). MEROPS: the database of proteolytic enzymes, their substrates and inhibitors. Nucleic Acids Res.

[26] Hahnke RL, Stackebrandt E, Meier-Kolthoff JP, Tindall BJ, Huang S, Rohde M (2015). High quality draft genome sequence of *Flavobacterium rivuli* type strain WB 3.3-2T (DSM 21788T), a valuable source of polysaccharide decomposing enzymes. Stand Genomic Sci.

[27] Rawlings ND, Barrett AJ, Bateman A (2012). MEROPS: the database of proteolytic enzymes, their substrates and inhibitors. Nucleic Acids Res.

[28] Fülöp V (1998). Prolyl oligopeptidase: an unusual beta-propeller domain regulates proteolysis. Cell.

[29] Polgár L (2002). The prolyl oligopeptidase family. Cell Mol Life Sci.

[30] Matos J, Nardi M, Kumura H, Monnet V (1998). Genetic characterization of pepP, which encodes an aminopeptidase P whose deficiency does not affect *Lactococcus lactis* growth in milk, unlike deficiency of the X-prolyl dipeptidyl aminopeptidase. Appl Environ Microbiol.

[31] Holst JJ, Deacon CF (1998). Inhibition of the activity of dipeptidyl peptidase IV as a treatment for type 2 diabetes. Diabetes.

[32] Rigolet P, Mechin I, Delage MM, Chich JF (2002). The structural basis for catalysis and specificity of the X-prolyl dipeptidyl aminopeptidase from *Lactococcus lactis*. Structure (London, England).

[33] Baba T, Schneewind O (1996). Target cell specificity of a bacteriocin molecule: a C-terminal signal directs lysostaphin to the cell wall of *Staphylococcus aureus*. EMBO J.

[34] Boos W, Eppler T, Winkelmann G (2001). Prokaryotic binding protein-dependent ABC transporters. Microbial transport systems.

[35] Boulanger A, De’jean G, Lautier M, Glories M, Zischek C, Arlat M (2010). Identification and regulation of the N-acetylglucosamine utilization pathway of the plant pathogenic bacterium *Xanthomonas campestris* pv. Campestris. J Bacteriol.

[36] He J, Ochiai A, Fukuda Y, Hashimoto W, Murata K (2008). A putative lipoprotein of *Sphingomonas* sp. strain A1 binds alginate rather than a lipid moiety. FEMS Microbiol Lett.

[37] Tang K, Jiao N, Liu K, Zhang Y, Li S (2012). Distribution and functions of TonB-dependent transporters in marine bacteria and environments: implications for dissolved organic matter utilization. PLoS One.

[38] Woese CR, Kandler O, Wheelis ML (1990). Towards a natural system of organisms: proposal for the domains Archaea, bacteria, and Eucarya. Proc Natl Acad Sci U S A.

[39] Garrity GM, Bell JA, Lilburn T, Garrity GM, Brenner DJ, Krieg NR, Staley JT (2005). Phylum XIV. *Proteobacteria* phyl. Nov. Bergey’s manual of systematic bacteriology. Volume 2, part B.

[40] Garrity GM, Bell JA, Lilburn T, BD GGM, Krieg NR, Staley JT (2005). Class I. *Alphaproteobacteria* class. Nov. second ed.

[41] Henrici AT, Johnson DE (1935). Studies of freshwater bacteria: II. Stalked bacteria, a new order of *Schizomycetes*. J Bacteriol.

[42] Skerman VDB, Gowan V, Sneath PHA, editors. Approved lists of bacterial names. Int J Syst Bacteriol. 1980;30:225–420.

[43] Henrici AT, Johnson D (1935). Stalked bacteria, a new order of *Schizomycetes*. J Bacteriol.

[44] Ashburner M, Ball CA, Blake JA, Botstein D, Butler H, Cherry JM (2000). Gene ontology: tool for the unification of biology. The gene ontology consortium. Nat Genet.

