# Supplementary material for: High-quality draft genome sequence of Aquidulcibacter paucihalophilus TH1–2T isolated from cyanobacterial aggregates in a eutrophic lake
Source: Stand Genomic Sci. 2017 Dec 2;12:69. doi: 10.1186/s40793-017-0284-9 (PMC5712168; doi:10.1186/s40793-017-0284-9)
Supplement: Additional file 1: Table S1. — comparison of proportions of COG categories between A. paucihalophilus TH1–2 T, E. coli K12, P. putida KT2440, and S. oneidensis MR-1. (DOCX 13 kb) [file 40793_2017_284_MOESM1_ESM.docx]

**Table S1. comparison of proportions of COG categories between *A. paucihalophilus* TH1-2^T^, *E. coli* K12, *P. putida* KT2440, and *S. oneidensis* MR-1**

| Name | TH1-2^T^ | K-12 | Kt2440 | Mr-1 |
| --- | --- | --- | --- | --- |
| General function prediction only | 8.85 | 10.06 | 11.51 | 10.36 |
| Translation, ribosomal structure and biogenesis | 7.12 | 4.69 | 3.92 | 5.40 |
| Amino acid transport and metabolism | 7.08 | 9.46 | 4.51 | 3.11 |
| Lipid transport and metabolism | 6.78 | 2.61 | 4.01 | 3.14 |
| Function unknown | 6.67 | 8.27 | 8.81 | 9.11 |
| Transcription | 6.56 | 7.61 | 9.22 | 7.10 |
| Cell wall/membrane/envelope biogenesis | 6.22 | 5.69 | 5.30 | 5.06 |
| Coenzyme transport and metabolism | 5.50 | 3.90 | 3.99 | 4.65 |
| Energy production and conversion | 5.20 | 7.40 | 6.30 | 6.94 |
| Carbohydrate transport and metabolism | 5.09 | 9.48 | 4.51 | 3.11 |
| Posttranslational modification, protein turnover, chaperones | 4.97 | 3.48 | 3.69 | 4.65 |
| Inorganic ion transport and metabolism | 4.90 | 5.61 | 6.07 | 4.99 |
| Signal transduction mechanisms | 4.22 | 4.43 | 6.00 | 6.72 |
| Replication, recombination and repair | 4.11 | 5.08 | 3.92 | 7.98 |
| Secondary metabolites biosynthesis, transport and catabolism | 3.92 | 1.61 | 2.72 | 1.63 |
| Intracellular trafficking, secretion, and vesicular transport | 2.90 | 3.08 | 2.31 | 2.83 |
| Defense mechanisms | 2.56 | 1.24 | 1.22 | 1.95 |
| Nucleotide transport and metabolism | 2.49 | 2.53 | 1.99 | 2.42 |
| Cell motility | 1.81 | 2.87 | 2.45 | 3.39 |
| Cell cycle control, cell division, chromosome partitioning | 1.13 | 0.84 | 0.77 | 1.13 |
| Chromatin structure and dynamics | 0.08 | 0.00 | 0.05 | 0.03 |
